# Comprehensive Study of Mechanical, Electrical and Biological Properties of Conductive Polymer Composites for Medical Applications through Additive Manufacturing

**DOI:** 10.3390/polym16182625

**Published:** 2024-09-17

**Authors:** Emese Paari-Molnar, Kinga Kardos, Roland Told, Imre Simon, Nitin Sahai, Peter Szabo, Judit Bovari-Biri, Alexandra Steinerbrunner-Nagy, Judit E. Pongracz, Szilard Rendeki, Peter Maroti

**Affiliations:** 1 3D Printing and Visualization Centre, University of Pecs, Boszorkany Str. 2, H-7624 Pecs, Hungary; paari.molnar.emese@pte.hu (E.P.-M.); kardos.kinga@pte.hu (K.K.); told.roland@pte.hu (R.T.); simon.imre@pte.hu (I.S.); maroti.peter@pte.hu (P.M.); 2 Medical Skills Education and Innovation Centre, Medical School, University of Pecs, Szigeti Str. 12, H-7624 Pecs, Hungary; 3 Department of Biomedical Engineering, North Eastern Hill University, Shillong 793022, Meghalaya, India; nitinbiomedical@gmail.com; 4 Institute of Geography and Earth Sciences, Faculty of Sciences, University of Pecs, Ifjusag Str. 6, H-7624 Pecs, Hungary; sz.piiit01@gmail.com; 5 Environmental Analytical and Geoanalytical Research Group, Szentágothai Research Centre, University of Pecs, H-7624 Pecs, Hungary; 6 Department of Pharmaceutical Biotechnology, Faculty of Pharmacy, University of Pecs, Rokus Str. 2, H-7624 Pecs, Hungary; bovari.judit@pte.hu (J.B.-B.); steinerbrunner.alexandra@pte.hu (A.S.-N.); pongracz.e.judit@pte.hu (J.E.P.)

**Keywords:** 3D printing, thermal analysis, mechanical analysis, electrical properties, polymer, carbon

## Abstract

Conductive polymer composites are commonly present in flexible electrodes for neural interfaces, implantable sensors, and aerospace applications. Fused filament fabrication (FFF) is a widely used additive manufacturing technology, where conductive filaments frequently contain carbon-based fillers. In this study, the static and dynamic mechanical properties and the electrical properties (resistance, signal transmission, resistance measurements during cyclic tensile, bending and temperature tests) were investigated for polylactic acid (PLA)-based, acrylonitrile butadiene styrene (ABS)-based, thermoplastic polyurethane (TPU)-based, and polyamide (PA)-based conductive filaments with carbon-based additives. Scanning electron microscopy (SEM) was implemented to evaluate the results. Cytotoxicity measurements were performed. The conductive ABS specimens have a high gauge factor between 0.2% and 1.0% strain. All tested materials, except the PA-based conductive composite, are suitable for low-voltage applications such as 3D-printed EEG and EMG sensors. ABS-based and TPU-based conductive composites are promising raw materials suitable for temperature measuring and medical applications.

## 1. Introduction

Additive manufacturing (AM) is a constantly evolving field [1,2]. According to ISO/ASTM 52900:2021 [3] standard, there are eight categories of AM processes. Commonly used AM technologies are laminated object manufacturing (LOM), material extrusion (MEX), vat photopolymerization (VPP), material jetting (MJT), and powder bed fusion (PBF) [4,5,6,7]. LOM is a process through which 3D objects are created using paper, plastic, and metals. In the MEX process, the materials are extruded through a nozzle and the objects are built layer by layer. Commercial MEX processes include fused filament fabrication (FFF), which is one of the most popular AM processes [8,9], and continuous fibres reinforcement (CFR). In CFR technology, the printer utilizes a second nozzle to lay down a continuous reinforcement of composite fibres within the FFF technology [10]. In the VPP process, a liquid photopolymer is selectively cured by light-activated polymerization in a vat. Stereolithography (SLA) is one of the most widely used variants of this technology. PBF is a process in which thermal energy selectively fuses regions of the powder bed. One of these technology groups is selective laser sintering (SLS). The MJT process involves droplets of feedstock material being selectively deposited. The PolyJet™ (manufactured by Stratasys, Ltd. (Rehovot, Izrael)) technology is an MJT process. The SLA, SLS, and PolyJet™ technologies are widely used in medical applications [11,12,13]

FFF technology can be used for concept design, modelling, and cost-effective low-volume production [8,9,14,15]. One possibility of this technology involves the use of 3D printing filaments with conductive additives such as carbon black or carbon nano tubes. To understand the thermoplastic behaviour of these composites, the mechanical and electrical properties of this type of printing materials were investigated.

Electrically conductive polymer composites offer a new alternative to traditional metal-based systems. There is a growing demand for flexible, soft, non-corrosive materials in the emerging fields of robotics, cybernetics, and biomedicine. Metallic fillers are less popular than carbon-based fillers because they often cause clogging problems during FFF printing [16]. Sensors measuring dynamic mechanical quantities, like force and acceleration, require conductive fibers with relatively high electrical resistance. Interconnections of electrical conductors can also be printed using FFF technology, but unlike sensors, these need to have relatively low specific resistance [8].

Several thermoplastic conductive composite filaments are already commercially available. The drawback is that the resistance of these filaments is 4–6 orders of magnitude higher than that of pure metal conductors, such as copper or silver [17]. By analysing the dielectric and thermal properties of twenty-four polymers used in 3D printing (FFF), it was found that, unlike injection moulding, 3D printed objects have structural homogeneity limitations that are strongly dependent on the moisture content of the filaments and the 3D printer setup, which can consequently affect the dielectric properties of the polymers [18]. The electrical and mechanical properties of conductive composites based on PLA and ABS have been investigated by Pentek et al. and Ujfalusi and co-workers. For use in prosthetics and the development of medical robotic devices, PLA-based and ABS-based conductive materials were found to be suitable [19,20]. Carbon nanotubes (CNT)/ABS composite filaments were prepared, and their conductivity was investigated. It was found that the CNT content significantly and non-linearly changed the mechanical and electrical properties of the ABS material [21].

Conductive polymers with carbon additives are frequently used as strain sensors and other low-voltage sensor types [22]. In one study, TPU/CNT flexible strain sensors with auxetic structure were invented. The sensors were manufactured via a hybrid method comprising FFF 3D printing and ultrasonic cavitation-enabled treatment [23]. Three types of periodic configurations for flexible strain sensors were designed. The periodic structures were manufactured from a composite of TPU and carbon black particles [24]. ABS/carbon black commercially available composite was tested for stress–strain gauge for health monitoring [25]. Different shapes of strain sensors were invented using neat TPU material and conductive PLA-based composite [26].

A wide range of medical applications, including electroencephalogram (EEG) sensors, electromyography (EMG) sensors, tactile sensors, and strain sensors, have been successfully developed and manufactured using 3D printing technology. Multi-channel EEG sensors for zebrafish were manufactured and developed using 3D printing by Cho et al. The 3D printed parts were deposited with Ti/Au composite [27]. In 2019, Velcescu and co-workers presented nine different configurations of flexible 3D printed EEG electrodes. The electrodes were coated with silver/silver chloride [28]. In 2020, 3D printable dry EEG electrodes with coiled-spring prongs were invented. The printed sensors were produced using an SLA printer [29]. In a previous study, EMG sensors were used in prostheses, and the signals were transmitted via 3D printed parts [20,30]. In 2021, researchers reviewed the use of polymers in sensors. e.g., for medical applications. The mechanical, electrical, and magnetic properties of the 3D printed sensors and specimens were studied. Printing settings have been found to have a major influence on the properties of the finished samples [31]. Yang et al. invented wearable flexible electronics. Flexible shape-memory devices have been coated with silver nanoparticles or carbon nanotubes [32]. Three-dimensional printed bionic ears have been invented by Mannor and coworkers. The ears were made from a chondrocyte-seeded alginate hydrogel matrix with electrically conductive silver nanoparticles and an inductive coil antenna and cochlea-shaped electrodes supported on silicone [33].

This paper investigates the mechanical, electrical, and thermal properties of conductive composite filaments based on PLA, ABS, TPU, and PA. In this study, the tensile, three-point flexural, Charpy impact, and Shore D hardness properties were measured. The resistance, signal transmissibility, and the relationship between resistance and temperature were also investigated. The relationship between cyclic elongation and resistance was examined. Scanning electron microscopy was performed on the Charpy tests’ broken specimens. Cytotoxicity tests were also carried out for each material. These tests are important in determining the suitability of the composite materials under investigation for use as medical sensors, including strain sensors, EEG and EMG sensors, and temperature and tactile sensors for use in prostheses. Strain sensors made of the matrix materials under investigation have been reported in the literature. The majority of these sensors are composites of a flexible substrate layer and a conductive layer in different shapes. The other type of strain sensor described in the literature is made of a single matrix composite with a special structure. Surprisingly, the simple structure tested in this study has not been investigated in previous work, only specialized single matrix composite structures. The aim of the study is to investigate whether the simple structure studied could be used as a sensor. Another aim of this work is the examination of the relationship between layer height and electrical resistivity for the composites tested.

## 2. Materials and Methods

**Figure 1:**
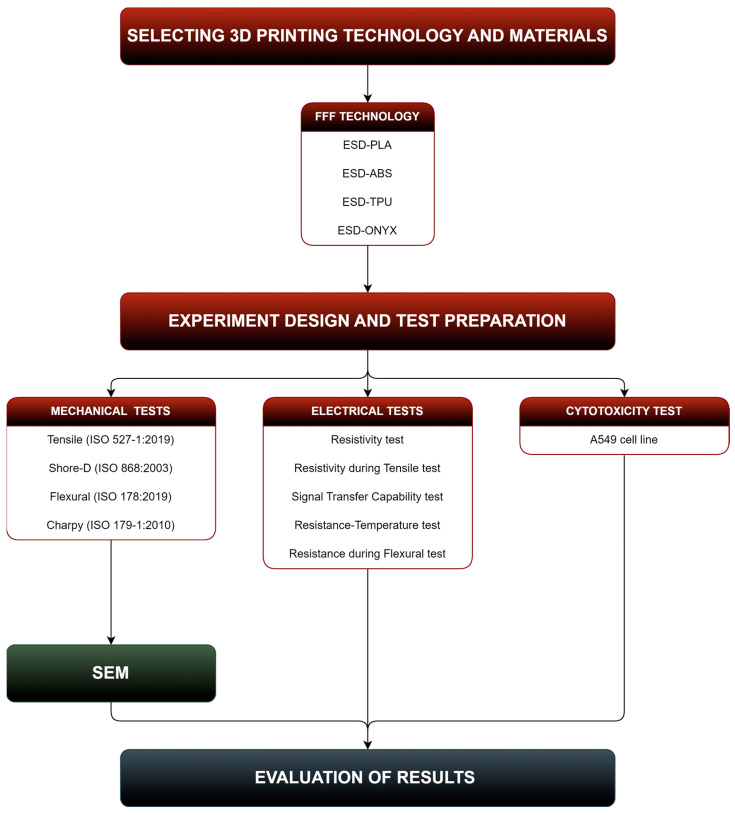
Method of the study.

The tested conductive polymers are based on PLA, ABS, TPU, and PA. The abbreviations of these composites in order are ESD-PLA, ESD-ABS, ESD-TPU, and ESD-Onyx (ESD, electrostatic discharge). For the fabrication of test specimens, FFF technology was used. The mechanical properties (tensile, flexural, Charpy, and Shore-D tests) and electrical properties (resistance, resistance during cyclic tensile tests, resistance during cyclically varying temperature tests, signal transfer capability) of the specimens were investigated. Scanning electron microscopy on Charpy specimens was carried out. The total number of specimens was 142. In Figure 1 the protocol diagram of the study is illustrated.

### 2.1. Materials and 3D Printing Parameters

The materials that were the subject of this research comprised the following conductive composites: ESD-PLA (Protopasta CDP11705; Protoplant Inc., 12001 NE 60th Way, Suite B-2,Vancouver, WA 98682, USA), a PLA-based composite with added carbon black; ESD-ABS (ABS Conductive; SUNLU, 92 Corporate Park STE C204 Irvine, 92606, CA, USA), an antistatic ABS filament; ESD-TPU (Conductive Filaflex Black; Recreus Industries, S.L. Polígono, Industrial Finca Lacy, 03600, Elda (Alicante), Spain), a flexible electrically conductive TPU filament filled with carbon black; and an ESD safe PA-based filament with chopped micro carbon fibers, ESD-Onyx (Markforged Onyx ESD; Markforged, 60 Tower Road, Waltham, MA, USA). The diameter of each filament was 1.75 mm.

All samples were produced using FFF technology. For the ESD-PLA, ESD-ABS, and ESD-TPU materials, a Craftbot Plus desktop 3D printer (Craftunique Ltd.; 1143 Budapest, Ilka Street 50, Hungary) was used; for the ESD-Onyx material, a Markforged X7^TM^ industrial CFR printer (Markforged; 60 Tower Road in Waltham, MA, USA) was utilized. All the specimens were printed in XYZ orientation according to ISO/ASTM 52900 standard.

All specimens were printed with 100% fill density, and for the mechanical tests, 200 µm layer height. The other printing parameters and layer heights for electrical tests of the investigated materials are shown in Table 1.

### 2.2. Mechanical Analysis

All the mechanical tests were repeated five times for each material, according to the relevant ISO standards. All tests were performed at an ambient temperature of 23 °C ± 1 °C.

#### 2.2.1. Tensile Test

Tensile testing was performed by a Zwick/Roell Z100THW universal test machine (ZwickRoell, 89079, Ulm, Germany). The tests were carried out according to ISO 527-1:2019 standard [34]. The specimens were 1A from the ISO 527-2:2012. The preload was set to 0.1 MPa; the testing speed was 1 mm/min for the determination of Young’s modulus, and was then set to 50 mm/min for the tests.

#### 2.2.2. Three-Point Flexural Test

The three-point bending test was carried out using the same Zwick/Roell Z100THW universal material tester. The tests were based on the ISO 178:2019 standard [35] with the preferred test specimen; its size was 80 mm × 10 mm × 4 mm. The preload was 0.1 MPa, and the testing speed was set to 2 mm/min during the full test. The support distance was 64 mm, and the maximal deformation was 4.7%, according to the standard.

#### 2.2.3. Charpy Impact Test

To measure the impact values, the specimens were tested using a Zwick/Roell HIT50P (ZwickRoell, 89079, Ulm, Germany) instrument utilizing a 5 J pendulum, following the ISO 179-1:2010 standard [36]. The size of the specimen was 80 mm × 10 mm × 4 mm. The edgewise impact was performed on the test specimen without notch.

#### 2.2.4. Shore D Hardness

The Shore D hardness tester was a Zwick/Roell 3131/320154 (Zwick/Roell, 89079, Ulm, Germany). The tests were performed according to ISO 868:2003 standard [37]. The instrument was set on a stable stand during the entire measurement process.

### 2.3. Electrical Measurements

#### 2.3.1. Electrical Resistance Measurements

Conductivity measurements were made out using a Voltcraft VC830 digital multimeter (Conrad Electronic SE, Klaus Condrad—Str. D-92240 Hirschau, Germany). Probes were placed at identical locations on the surface of bolt nuts, which were placed on the screws at the end points of the specimens. This method was used for the initial resistance measurements in the cyclic tensile and temperature tests.

#### 2.3.2. Resistance Measurement during Tensile Test

**Figure 2:**
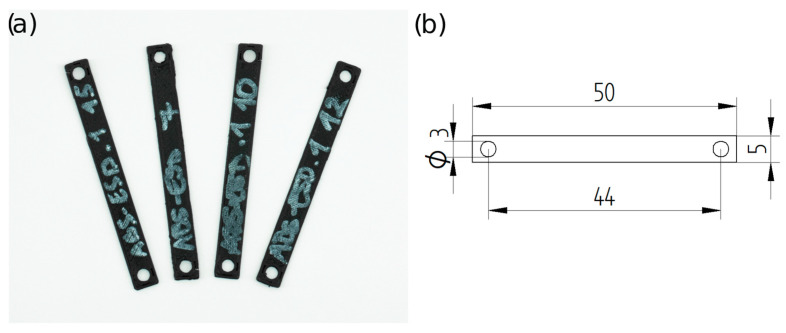
(**a**) Photograph (ESD-ABS). (**b**) Schematic representation of the specimens for the tensile–resistance test.

The resistance of the specimens was measured during tensile tests. For the measurement, the same Zwick/Roell Z100THW universal test machine was used, and the resistance was measured throughout the test with an Arduino Uno R1 (Arduino srl., Arduino.cc). The specimens’ dimensions were 50 mm × 5 mm × 0.6 mm, with 3 mm holes at the ends (Figure 2). First, the specimens were stretched for 10 cycles, and then subjected to a tensile test.

The four materials were tested with four different stretching modes. The strain was set between 0.3 mm and 0.65 mm for ESD-PLA, 0.2 mm and 0.8 mm for ESD-ABS, 0.1 mm and 0.5 mm for ESD-TPU, and 0.7 mm and 1.1 mm for ESD-Onyx. The relationship between the maximum resistance per cycle and the elongation was examined.

The gauge factor of a strain gauge is the ratio of the relative resistance change and relative length change. It is defined as:

(1) $$GF=\frac{\frac{R-R_{0}}{R_{0}}}{\varepsilon },$$

where R is the measured resistance during the tensile test [Ω], R_0_ is the initial resistance [Ω], and ε is the strain during the tensile test [%].

#### 2.3.3. Resistance–Temperature Measurement

**Figure 3:**
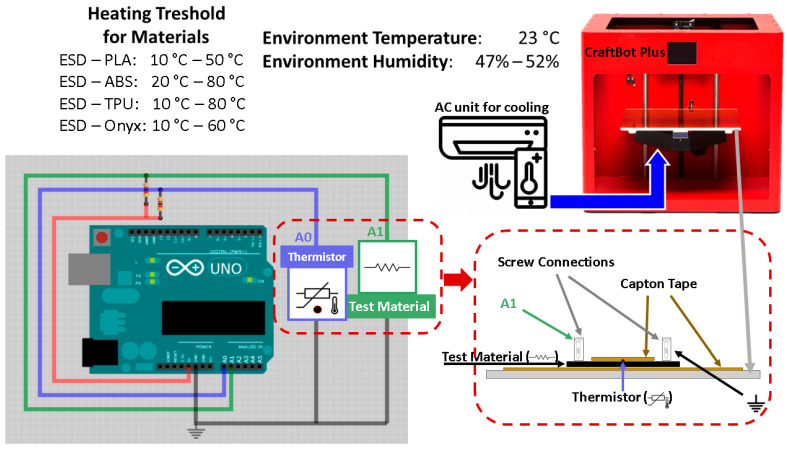
Measuring equipment for temperature–resistance measurements. The specimen was insulated and fixed to the printing bed with Kapton tape. On the top, between the tape and the specimen, a thermistor was inserted, and the sample was connected to the voltage divider on both sides.

The relationship between the thermal and electrical properties of the composite materials was investigated. The size of the specimens was 50 mm × 5 mm × 0.6 mm. The measurements were repeated for each specimen after 24 h. The measurement was carried out with Arduino Uno R1 as a resistance meter and the heated print bed of a Craftbot Plus desktop 3D printer as a heater. Temperature was measured using a thermistor with 100 kΩ resistance. Kapton tape was used for insulation and fixing of the specimens to the printing bed of the 3D printer. The temperature interval was varied between 10 °C and 50 °C for ESD-PLA, 20 °C and 80 °C for ESD-ABS, 10 °C and 80 °C for ESD-TPU, and 10 °C and 60 °C for ESD-Onyx composites. The sampling frequency was fs = 10 Hz. The measuring equipment (Figure 3) was identical to the resistance–temperature measurement setup used by Ujfalusi et al. [20].

#### 2.3.4. Resistance during Flexural Test

**Figure 4:**
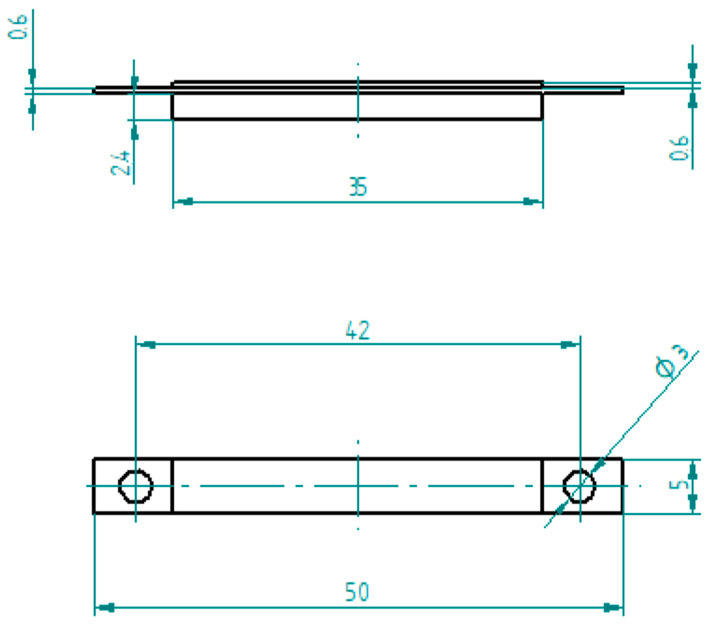
Schematic representation of mixed flexural specimen.

The effect of bending the conductive composite on the resistance was investigated. The specimens (mixed flexural specimens) were made from a neat polymer and a composite material. The schematic drawing of the specimen is in Figure 4. The conductive polymer was surrounded from the bottom and top by the neat polymer, ensuring that the whole of the conductive part remained in the bending zone. The length of the neat polymer parts (35 mm) was shorter than the conductive polymer part (50 mm). The whole specimen had a thickness of 3.6 mm, the conductive part had a thickness of 0.6 mm, and the neat parts had a thickness of 1.8 mm and 0.6 mm. The investigation was implemented using the same Zwick/Roell Z100THW universal material tester for bending the specimens and an Arduino Uno as a resistance meter.

#### 2.3.5. Signal Transfer Capability

**Figure 5:**
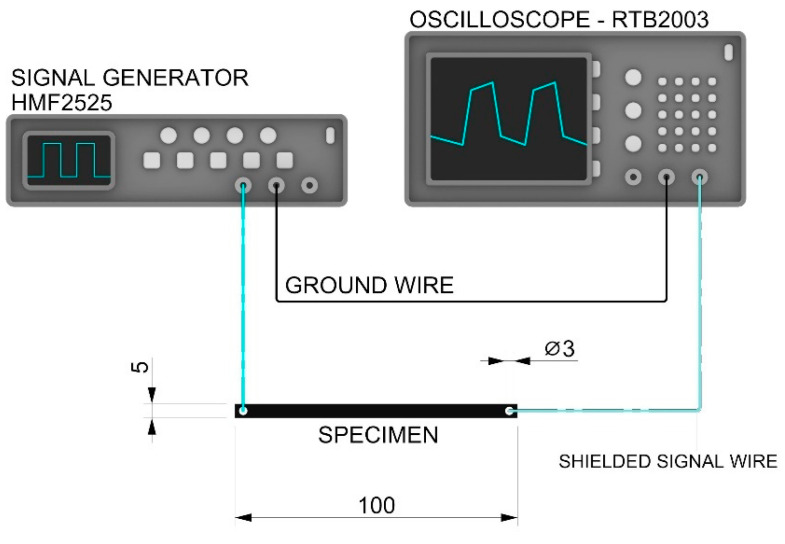
Schematic representation of the signal transfer measurements.

To determine the signal transfer, specimens measuring 100 mm × 5 mm × 1.2 mm with 3 mm holes at the ends were prepared (Figure 5) and a Rohde & Schwarz HMF2525 25 MHz function generator and a Rohde & Schwarz RTB2003 2.5 Gsa/s digital oscilloscope (Rohde & Schwarz USA, Inc. 6821 Benjamin Franklin Drive, Columbia, MD 21046, USA) were used.

Excitation sinus-signals were transferred in the X direction of specimens, with different frequencies that varied between fe = 100 Hz and 500 kHz. The test was performed between 100 Hz and 500 kHz for the ESD-PLA, ESD-TPU, and ESD-Onyx composites, and between 100 Hz and 250 kHz for the ESD-ABS composite. The amplitude was set U_e_ = 1 V in the cases of the ESD-PLA, ESD-ABS, and ESD-TPU, and U_e_ = 10 V for ESD-Onyx.

### 2.4. Scanning Electron Microscopy (SEM)

A JEOL JSM-IT500HR SEM (3–1–2 Musashino, Akishima, Tokyo 196–8558, Japan) was used for SEM tests. For this purpose, the Charpy specimens’ broken surfaces and the intact surfaces of the test bars were scanned with 250×, 1000×, and 15,000× magnification, and Markforged matrix and carbon material with 2000×. Before imaging, the samples were coated with gold using a JEOL JFC-1300 auto-fine coater.

### 2.5. Cytotoxicity

#### 2.5.1. Cell Culturing and Treatment

A549 cells (ATCC, Manassas, VA, USA, Cat.no: CRM-CCL-185) were cultured in Dulbecco’s modified Eagle’s medium (Lonza, Belgium, Cat. no: 12604F)) at 37 ˚C, in a humidified atmosphere containing 5% CO_2_. The medium was supplemented with 10% foetal bovine serum (FBS, Euroclone, Italy, Cat. no.: ECS0180L), 100 U/mL of penicillin–streptomycin (Lonza, Belgium, Cat. no.: DE-17-602E), 0.1 mM non-essential amino acids (Lonza, Belgium, Cat. no: 13-114E), 10 mM Hepes (Lonza, Belgium, Cat. no: 17-737E), and diluted ß-mercaptoethanol (Thermo, Waltham, MA, USA, Cat. no: 21985–023). All the cell viability tests were performed in triplicates and repeated in three independent experiments (n = 9). The tested inserts made from various polymer materials were placed into the wells of a 24-well plate. To prevent the contamination of the cell cultures, the surfaces of the tested inserts were UV irradiated for 2 × 30 min on both sides.

A549 cells were resuspended in 1 mL DMEM medium, seeded to the surface of a 24-well plate at 3 × 10^4^ cell/well density, and incubated for 8 h at 37 °C in a humidified atmosphere containing 5% CO_2_. Once the cells were attached to the surface of the cell culture plate, the printed synthetic inserts were placed into each well and cultured for 48 h. A549 cells treated with 1 µM staurosporine (Sigma Aldrich, Burlington MA, USA, Cat. no: S-6942) were used as positive controls for the detection of apoptotic cell death in the total nucleic acid content determination assay.

#### 2.5.2. Light Microscopy

Light microscopic images were taken by a Nikon Eclipse light microscope using a 20× magnification. Scale bars were layered on the images using ImageJ 1.46 software.

Living cell number quantification was performed based on total nucleic acid content determination.

An applied cell viability assay was conducted based on the detected fluorescence intensity of propidium iodide (PI) (Sigma-Aldrich, Cat. no: 81845) staining. The approach was different from the standard PI staining protocols. After 48 h exposure to the synthetic inserts (ESD-ABS, ESD-PLA, ESD-TPU, and ESD-Onyx), total nucleic acid content was determined by PI staining on the Perkin-Elmer EnSight Multimode plate reader. Briefly, the treated cells were washed in 500 µL of PBS (phosphate buffered saline) (pH 7.4) to remove all the detached apoptotic cells. After removing apoptotic cells, adherent, living cells were lysed in 500 μL cell-lysing borate buffer (pH 9.2) and labelled with PI at 2 μg/mL concentration. After a 5-min incubation on an orbital shaker in a dark environment, fluorescent intensities were measured at λ excitation = 530 nm and λ emission = 620 nm wavelengths. All the treated sample results were compared to the untreated controls.

The analysis of the cytotoxicity results was generated in GraphPad 9 Software. Statistical analysis was performed by one-way ANOVA with the Dunnet post hoc test after data passed the normality test using the Kolmogorov–Smirnov test. Significance levels were labelled as follows: non-significant (ns), *p* < 0.0001 (****) [38].

### 2.6. Statistical Analysis

The measurement data were assessed and visualized using Origin Pro 2018 (OriginLab Corporation, One Roundhouse Plaza, Suite 303, Northampton, MA 01060, USA). The results of the cyclic tensile tests and resistance–temperature measurements were smoothed using the 15 pts Adjacent Averaging smooth method of the Origin Pro 2018. For the resistance measurement during tensile test, the relationship between the maximum resistance per cycle and the elongation was evaluated using a fitted curve. The fitted function was as follows:

(2) $$y\left(x\right)=A_{1}e^{\frac{x}{t_{1}}}+y_{0,}$$

where A_1_ is the amplitude (constant) [Ω], t_1_ is a constant [mm], and y_0_ is the offset [Ω]. In this case, x is the elongation [mm], and y is the fitting curve of the maximum resistances as a function of elongation [Ω]. The limit of the fitting function is y_0_ [Ω]. The fit is considered good if the R^2^ parameter is above 0.97.

## 3. Results

### 3.1. Mechanical Tests

**Figure 6:**
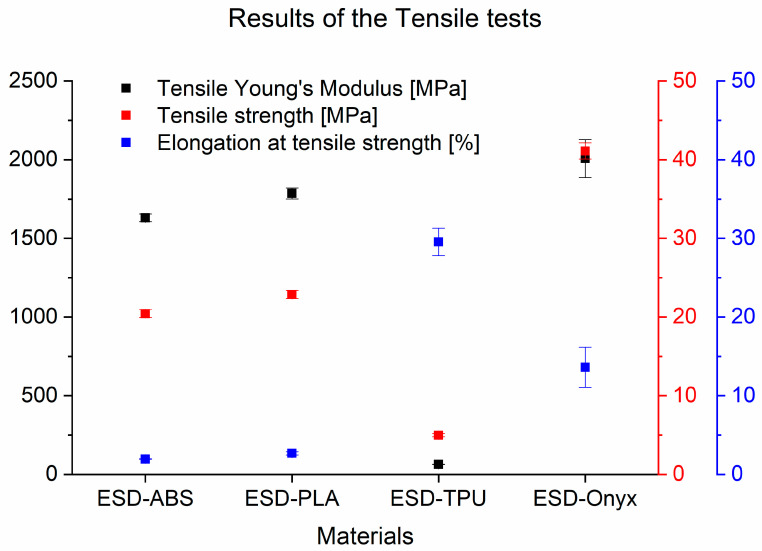
Results of the tensile tests of ESD-ABS, ESD-PLA, ESD-TPU, and ESD-Onyx.

Figure 6 presents the experimental data from tensile tests. It clearly shows that the most rigid composite material was ESD-Onyx with a tensile Young’s modulus of 2006 MPa ± 120.7 MPa and a tensile strength of 41.10 MPa ± 1.02 MPa. The ESD-TPU samples had the lowest tensile Young’s modulus (63.72 MPa ± 2.09 MPa) and tensile strength (4.98 MPa ± 0.21 MPa), but elongation at tensile strength was the highest for these specimens with a value of 29.56% ± 1.73%. In Table 2, there is a summary of the results of the tensile tests of the composites.

**Figure 7:**
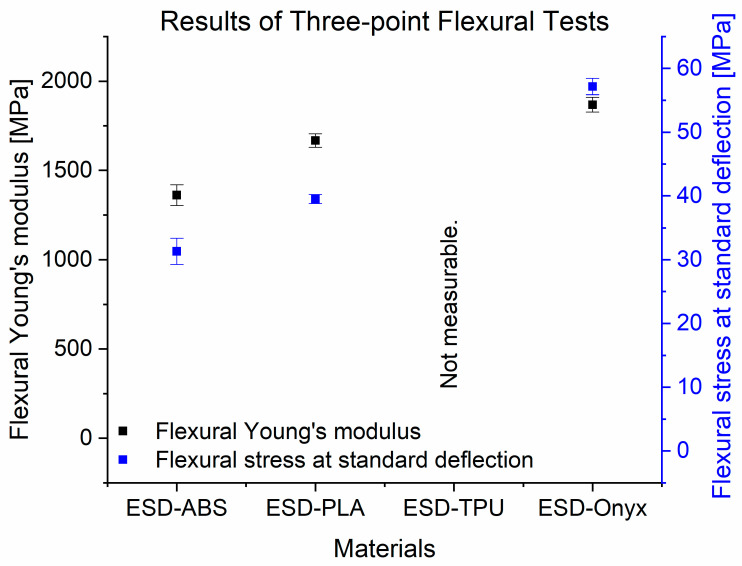
Results of three-point bending tests of all tested materials.

In Figure 7, the experimental data from the three-point flexural tests for all materials tested are displayed. The ESD-Onyx samples also had the highest flexural Young’s modulus (1868 MPa ± 41.83 MPa) and flexural stress at conventional deflection (57.16 MPa ± 1.29 MPa). ESD-ABS had the lowest flexural Young’s modulus and flexural stress at standard deflection.

**Figure 8:**
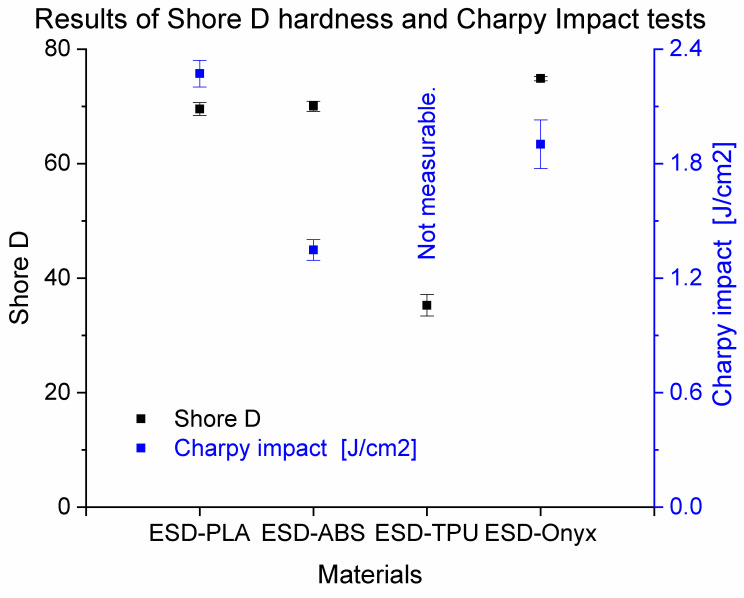
Experimental data from the Shore D hardness and Charpy Impact tests for all tested materials.

Figure 8 shows the results of the Shore D hardness and Charpy impact tests for all materials tested. The ESD-Onyx composite showed the highest value of Shore D hardness (74.86 ± 0.38). The lowest Shore D hardness was also observed for ESD-TPU materials. The highest Charpy impact strength was observed for the ESD-PLA composite, with a value of 22.7 kJ/m^2^ ± 0.7 kJ/m^2^, but ESD-Onyx also had a similar impact strength (19 kJ/m^2^ ± 1.3 kJ/m^2^). The Charpy impact was not measurable for the ESD-TPU composite material due to its flexibility. All measured values can be found in the Supplementary Materials in the mechanical_sum.xlsx file.

### 3.2. Electrical Tests

#### 3.2.1. Resistance Measurement during Tensile Test

The resistance during the cyclic tensile test was measured for all the composites tested. In the cases of the ESD-PLA and ESD-Onyx composites, there was no definite relationship between the electrical resistance and the force, and neither the strain.

**Figure 9:**
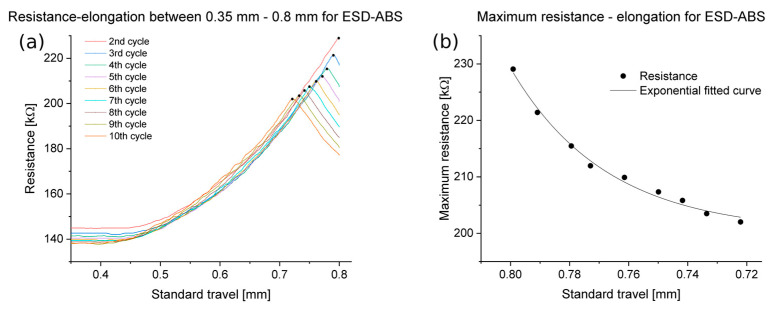
(**a**) Resistance, standard travel for an ESD-ABS specimen with 100 µm layer height, the maximums were marked with black dots. (**b**) Maximum resistance, standard travel for the same specimen. The black dots indicate the maxima per cycle and the line indicates the fitted curve.

The results indicated that elongation below 0.35 mm did not significantly affect the resistance in the case of ESD-ABS. The complete set of measurements is depicted in the abs_all_cycles_tensile.png file, which can be found in the Supplementary Materials. Figure 9 shows the relationship between the resistance and the elongation for an ESD-ABS specimen with 100 µm layer height. The graph only shows the range of 0.35 mm to 0.8 mm of standard travel, with the first cycle being a setup cycle, not presented in the graph. Subsequent cycles exhibited identical behaviour. The maximum resistance per cycle is marked with black dots. Figure 9b shows the relationship between the strain and the maximum resistance per cycle for the same specimen as in Figure 9a. The fitted function is represented by the black line on the same figure. At the higher layer heights of 200 µm and 300 µm, the maximum resistances during cycles may not always be monotonic. However, in cases where the maximum resistances exhibit monotonicity, the exponential fit was successful. The gauge factor was between 30 and 50.

**Figure 10:**
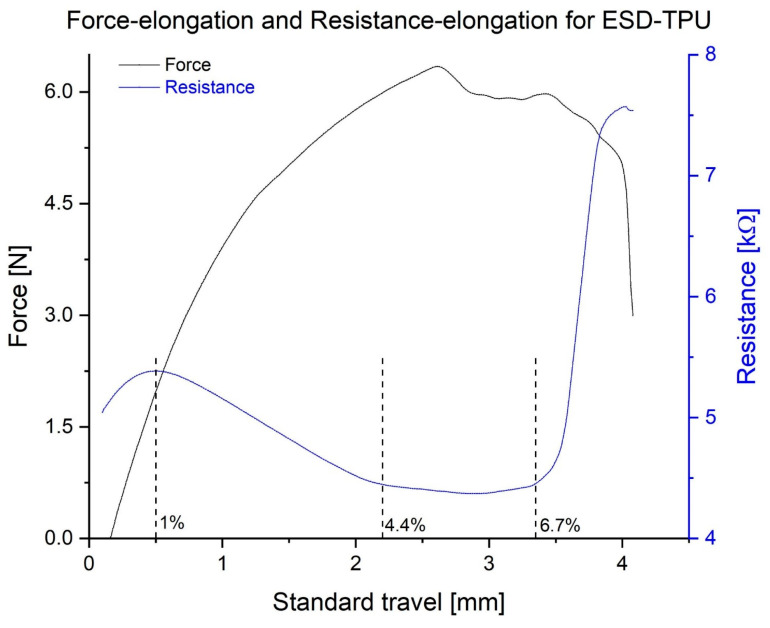
Standard force and electrical resistance in elongation curve in case of the ESD-TPU specimen with 200 µm layer height. Dashed lines indicate the boundaries of the sections for the different gauge factors, and the percentage values show these elongation limits.

The ESD-TPU composite specimens exhibited an interesting behaviour: their electrical resistance was not monotonic in the elongation curve. In Figure 10, a plot showing the tensile force and resistance as a function of the elongation is displayed. The resistance decreases after an initial increase, then increases suddenly before the complete rupture. This behaviour was common for all specimens printed from ESD-TPU.

The gauge factor for this case is GF = 8.5 (0.55% < ε < 1%) for the first increasing stage, GF = −6.1 (1% < ε < 4.4%) for the decreasing stage, GF = −0.18 (4.4% < ε < 6.7%) for the nearly constant phase, and GF = 65 (6.7% < ε < 8.2%) for the second and final increasing stage.

**Figure 11:**
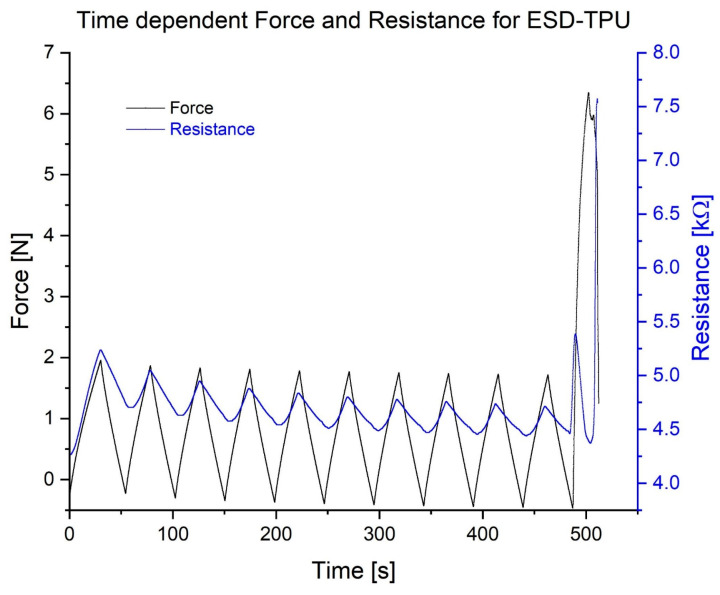
Tensile force and electric resistance as a function of time in the case of ESD-TPU specimen with 200 µm layer height.

Figure 11 shows the resistance and tensile force as a function of time for the same specimen as in Figure 10. There is a delay in the minimum resistance compared to the unstretched condition for all the specimens tested. The peaks of force and resistance are synchronized.

#### 3.2.2. Resistance–Temperature Measurement

**Figure 12:**
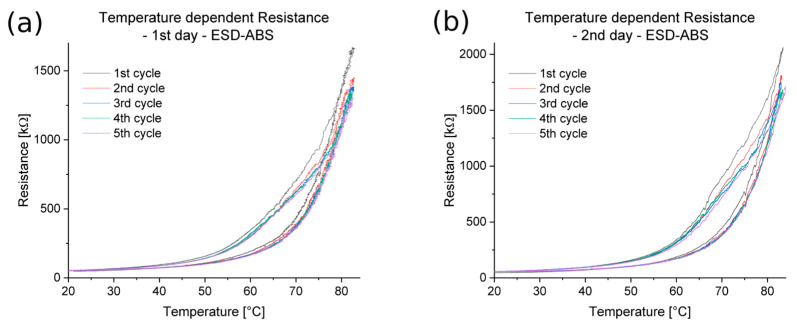
Temperature–electrical resistance relationship for an ESD-ABS specimen with 200 µm layer height. (**a**) First day measurement and (**b**) repeated, second day measurement.

In the cases of ESD-PLA specimens, the temperature and electrical resistances did not show a clear relationship. However, for the ESD-ABS specimens, the relationship was noticeable, as shown in Figure 12. According to the measurements, there was no point in investigating this relationship below 20 °C. The first and the repeated measurements had similar plots, but in the second one, there were higher values.

**Figure 13:**
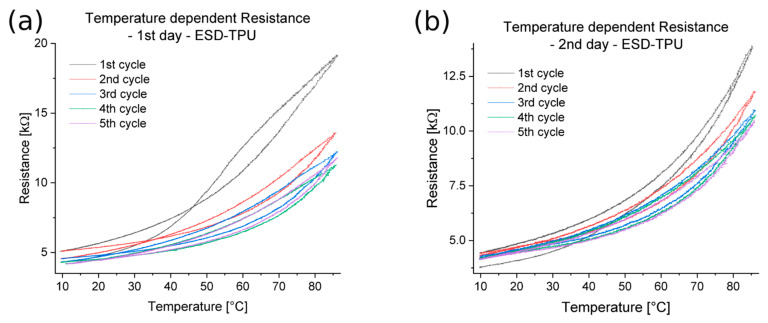
Temperature–electrical resistance relationship for an ESD-TPU specimen with 200 µm layer height. (**a**) First measurement and (**b**) repeated, second measurement.

Figure 13 shows the relationship between the temperature and electrical resistance for a specimen from the ESD-TPU composite with 200 µm layer height. On the plots it is illustrated that the first measurement for this composite is different from the second, and was repeated after 24 h. In the first measurement (Figure 13a), the graph of the cooling phase intersected the graph of the heating phase. In the cooling phase, the resistance was lower than the resistance in the heating phase at low temperatures. In the case of the repeated measurements (Figure 13b), the heating and cooling phases were kept at the same resistance at the low temperature interval. The graphs illustrate that the first cycle differed from the following ones. For all the specimens tested from this composite, the first cycle of the first measurement was a setup cycle, while the other cycles (both the first measurement and the repeat measurement) were in the same range.

The relationships between the temperature and the electrical resistance for the ESD-Onyx specimens have shown that maximum resistances occur at 20 °C ± 2 °C, and a decrease occurs above this temperature range.

#### 3.2.3. Resistance Measurement during Flexural Test

Resistance measurements were carried out using three-point flexural tests. The specimens were mixed from native and conductive polymers of the same type. ESD-ABS and ESD-TPU materials were examined with this type of examination.

**Figure 14:**
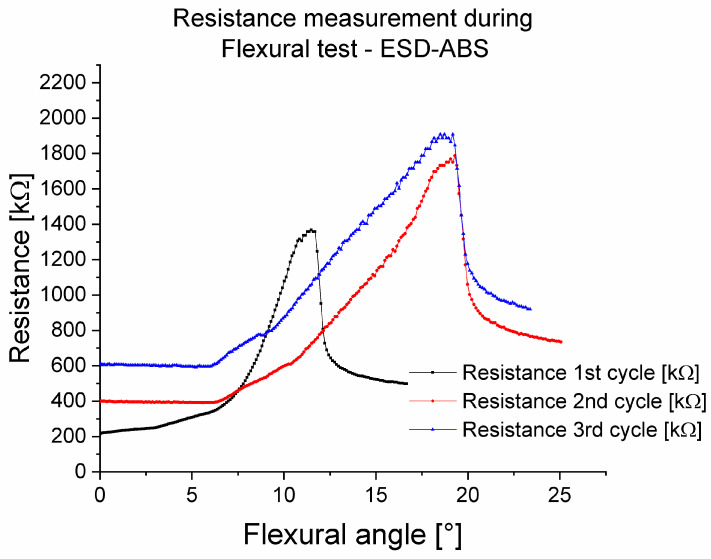
Resistance during flexural test in the case of the ABS-ESD-ABS mixed specimen.

Figure 14 shows the resistances during flexural tests in three cycles for the ABS-ESD-ABS mixed specimen with 200 µm layer height. The measured results show that the first stretching cycle was a so-called set-up cycle. This has also been observed in other examinations in this work. In the other two cycles, the flexural angle–resistance relationship was linear between 8° and 18°.

**Figure 15:**
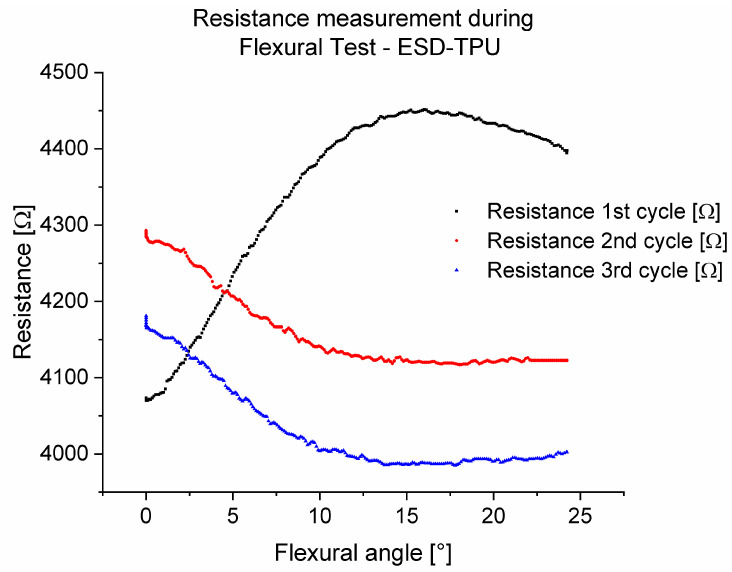
Resistance during flexural test in the case of TPU-ESD-TPU mixed specimen.

In Figure 15, the resistances during flexural tests in three cycles for the TPU-ESD-TPU mixed specimen with 200 µm layer height are presented. As previously shown, in this investigation, the ESD-TPU composite showed a set-up cycle, and after that, with the growth of the flexural angle, the resistance decreased, and after 17°, the resistance increased.

#### 3.2.4. Signal Transfer Capability

**Figure 16:**
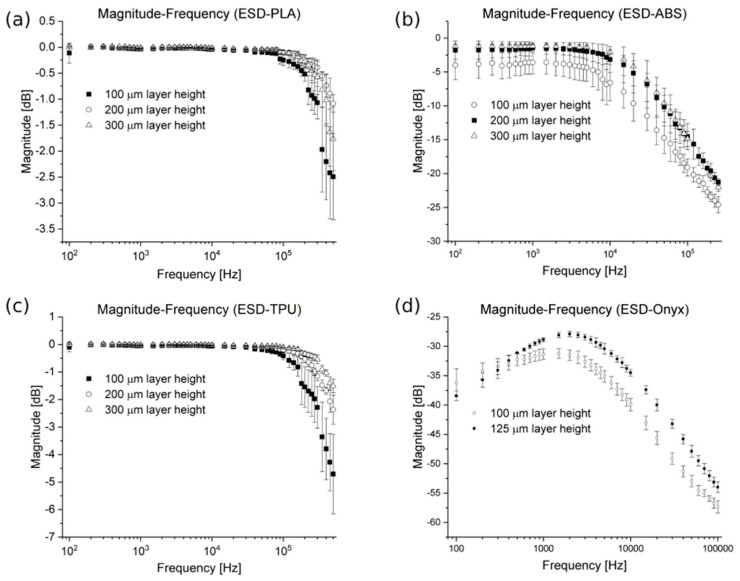
Bode plots with attenuation and standard deviation. ESD-PLA (**a**), ESD-ABS (**b**), ESD-TPU (**c**), and ESD-Onyx (**d**).

To examine the signal transfer capability, sinus waves were sent through the specimens. Figure 16a–d visualize the results of the signal transmission capability on Bode plots.

The attenuation stayed below 2 dB up to 300 kHz for the ESD-PLA material (Figure 16a). In the case of the ESD-ABS specimens, the attenuation stayed below 2 dB up to 15 kHz for the 200 µm and 300 µm, and the attenuation and the standard deviation differed for 100 µm (Figure 16b). For the ESD-TPU samples, the attenuation stayed below 2 dB up to 150 kHz (Figure 16c) for all layer heights. For ESD-Onyx there was no appreciable attenuation observed. (Figure 16d). At an input voltage of 10 V, the maximum measured voltage for ESD-Onyx was only 286 mV.

### 3.3. Scanning Electron Microscopy

**Figure 17:**
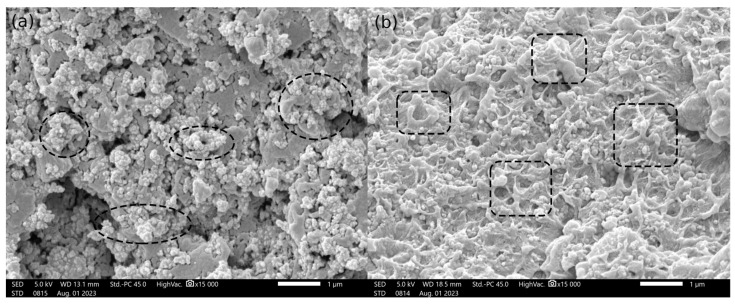
SEM images of the fracture surface of (**a**) ESD-PLA, (**b**) ESD-ABS at 15,000× magnification (scale bar is 1 µm). The black dashed ellipses indicate the carbon black granules and black dashed rectangles show the carbon nanotubes.

Scanning electron microscopy was performed on the fracture surface of Charpy specimens for all tested materials. Figure 17 shows SEM images of ESD-PLA (Figure 17a) and ESD-ABS (Figure 17b) at 15,000× magnification. From the ESD-TPU composite there was no valuable SEM image.

The conductive material of the ESD-PLA and ESD-TPU composites was carbon black. The SEM image of the ESD-PLA shows the granules (marked with white dashed ellipse). According to the SEM image of the ESD-ABS sample (Figure 17b), the conductive material in this composite could be carbon nanotubes (marked with black dashed rectangles).

**Figure 18:**
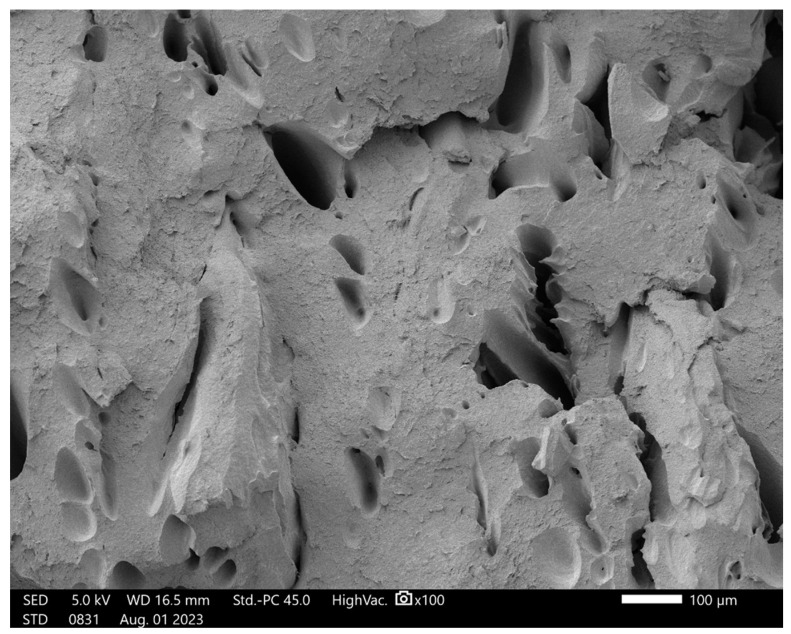
SEM image of the fracture surface of ESD-TPU at 100× magnification.

Figure 18 represents the fracture interface of an ESD-TPU specimen at 100× magnification. Holes can be observed on surface. The size of the holes was very variable, but in general, they were comparable to the thickness of the layer (200 µm). The material had not fused properly.

**Figure 19:**
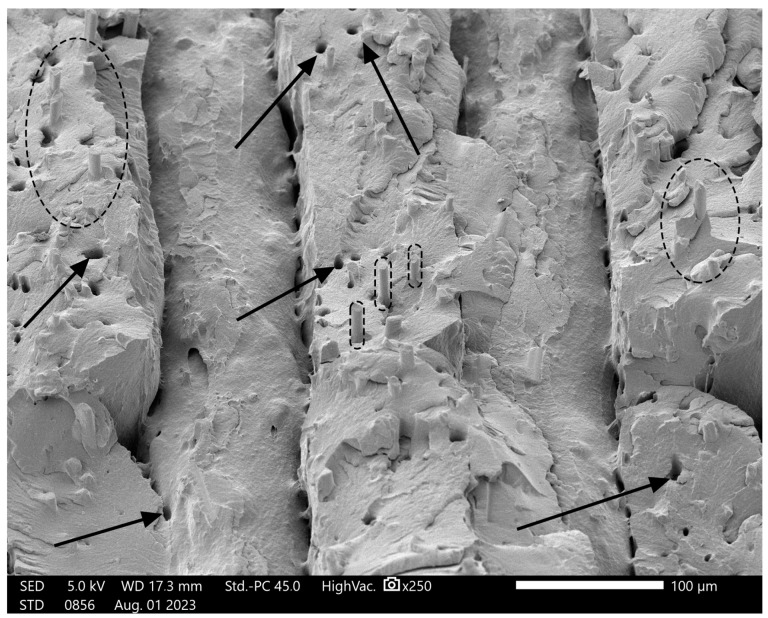
Fracture surface of ESD-Onyx material at 250× magnification. The black dashed ellipses indicate micro carbon fibres, and arrows show holes on the surface.

Figure 19 shows the fracture interface of the ESD-Onyx at 250× magnification. There are tubes visible (marked with dashed lines). These shapes could be the micro carbon fibres in the composite. On the surface, there are also holes (marked with arrows). Holes and rods complement each other. The periodic structure of the layers in the fracture surface is illustrated. As the figure shows, the layers have not really fused. This can be caused by 3D printing at the wrong temperature, but we have no control over these settings.

SEM images of Markforged Onyx and Markforged ESD-Onyx were compared. The result of the comparison and the comparative SEM images can be found in the Supplementary Materials (supplementary.docx).

### 3.4. Cytotoxicty

#### 3.4.1. Light Microscopy

**Figure 20:**
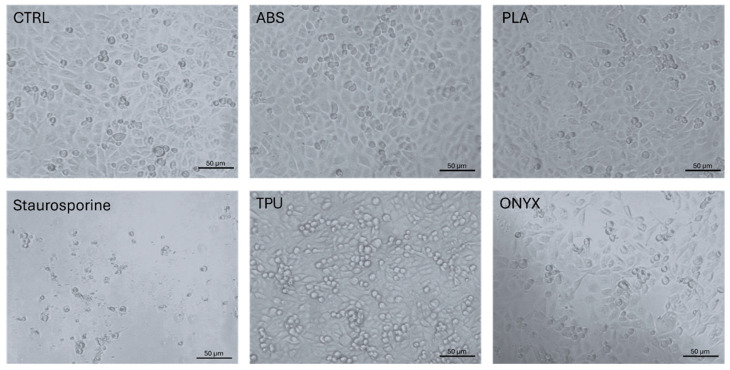
Light microscopic images of A549 cell lines following a 48 h incubation in the presence of various 3D printed materials (magnification: 20×, scale bar: 50 µm).

Light microscopic analysis showed no relevant changes in A549 cell line confluency following the 48-h incubation in the presence of the 3D-printed inserts. The cell confluency of the ESD-ABS, ESD-PLA, ESD-TPU, and ESD-Onyx-treated samples was close to the confluence of the untreated control sample, while staurosporine drastically decreased the viable cell number (Figure 20).

#### 3.4.2. Cytotoxicity

**Figure 21:**
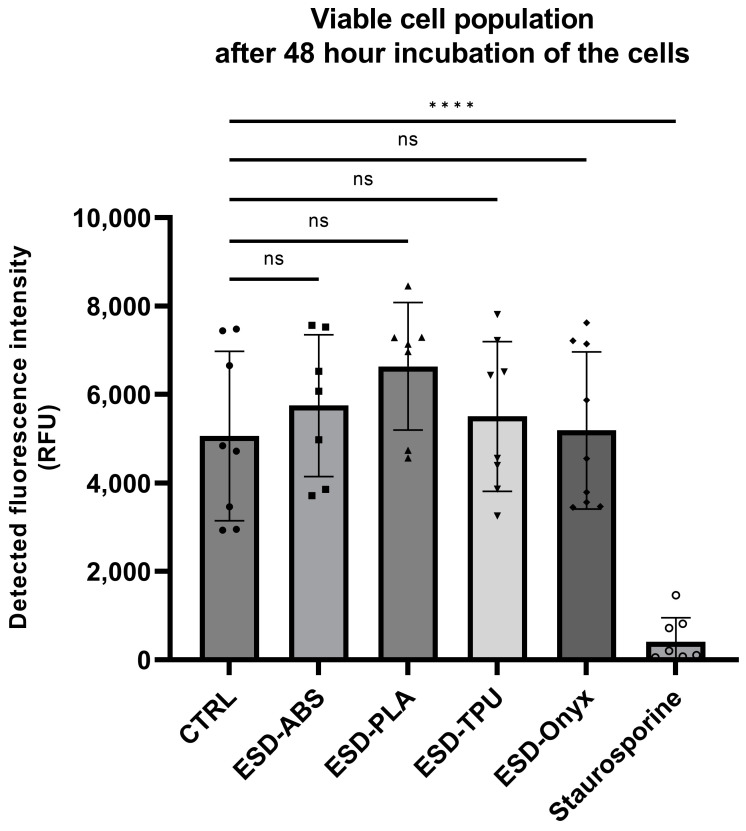
Quantification of the living cell number after 48 h incubation of A549 cells in the presence of various 3D-printed inserts (statistical analysis was performed in GraphPad 9 software, using one-way ANOVA with Kolmogorov–Smirnov normality test, n = 9, error bars represent SD, significance levels were labelled according to the following: *p* < 0.0001 (****) "ns" indicates that the bias was not significant.

The synthetic 3D printed inserts did not show cytotoxic effects on the cells of the A549 cell line after a 48 h incubation period. A slight increase in cell viability was detected using inserts made from ESD-PLA, ESD-ABS, and ESD-TPU (in order); however, the difference in cell number was not statistically significant compared to the untreated control samples (Figure 21).

## 4. Discussion

Conductive polymers are widely used in medical applications and low-voltage solutions. This study investigated the mechanical and electrical properties of conductive polymer samples with carbon-based additives. The composites tested were based on PLA, ABS, TPU, and PA. Mechanical tests included tensile, three-point flexural, Charpy impact and, Shore-D hardness tests. Electrical tests consisted of resistance tests, resistance measurement during cyclic tensile tests, resistance during cyclical variation of temperature tests, resistance during flexural tests, and tests of the signal transmission capability. On the Charpy specimens, scanning electron microscopy was performed. These measurements are necessary to determine whether the composites tested can be used in medical applications such as EEG and EMG sensors and tactile and strain sensors in prosthetics.

Considering the results of the mechanical tests, the most rigid specimens were made of the PA-based, ESD-Onyx composite. The most flexible samples were made of ESD-TPU material.

Based on the electrical measurements, the resistance of the specimens depended on the layer height; if it was greater, the resistance was lower for all tested composites. This relationship was not observed in the case of the ESD-TPU and ESD-Onyx. It was also confirmed by Péntek and coauthors [19] for similar PLA-based and ABS-based composites with carbon-based additives from another manufacturer; in that paper, only the resistance–temperature relationship was measured.

The change in resistance during the cyclic tensile tests was investigated for all composite materials. The tested composite materials had the property that the electrical resistance changes with the tensile force. The ESD-ABS composite had predictable behaviour in cyclic tensile tests. The relationship between the resistance and the tensile force was clear in a cycle. The maximum of the resistance per cycle exhibited exponential decreasing behaviour. The gauge factor of the specimens from ESD-ABS was relatively high [39]. This material, with its simple structure, had good strain properties. The examined gauge factor of the ESD-ABS specimens, ranging from 0.35 mm to 0.8 mm elongation, was higher than that found in the literature. The specimens from this composite material with 100 µm layer height can be applied for testing the durability and investigating the failure mechanics and strength of a material in various electronic devices. ESD-ABS could be a future printed material that is applied in various medical devices like flexible pressure sensors, surgical robots, implants, medical imaging, and piezo-resistive sensing devices for prosthetics [31,40,41,42]. It could also have various extended applications in healthcare industries, as it measures stress–strain tissues during surgery, early detection of stiffness in tissues, stress–strain on prosthetic joints, and early identification of pressure ulcers in patients. Taking into account the above results, fabricating an auxetic structure from this conductive ABS composite, like [23,24] suggested, could result in better strain properties than in the results presented.

During cyclic tensile testing, the ESD-TPU samples exhibited specific behaviour. The resistance of the specimens was not monotonic during elongation. After a short increasing phase, the resistance decreased in the elongation phase. In this phase, the ESD-TPU samples had a negative gauge factor, which could be explained by the Poisson factor [43,44]. The thickness of the specimen decreased as it stretched, bringing the conductive particles closer together and reducing the resistance. In the cyclic tensile tests, the resistance minima were delayed in relation to the force minima, whereas the maxima coincided for ESD-TPU. This was probably due to the regeneration time of the polymer molecule structure. ESD-PLA and ESD-Onyx composites were also investigated using this test type, but the data showed no valuable results.

The behaviour of the resistance during flexural tests was examined with mixed specimens from neat and conductive polymers for the cases of ESD-ABS and ESD-TPU. Both conductive composites were subjected to a set-up cycle. After this cycle, the resistance was a linear function of the angle between 8°and 18° for ESD-ABS. In the case of ESD-TPU, the resistance decreased with the flexural angle, which could also be due to Poisson’s ratio, as in the case of the resistance–tensile tests.

The relationship between temperature and electrical resistance was investigated for all tested composites. It can be concluded that the ESD-ABS and ESD-TPU materials could be used as raw materials for thermometers. This property has been investigated by Ujfalusi and coworkers for similar ABS-based materials [20]. For both materials, the electrical resistance was a monotonic function of temperature during heating and cooling. During the tests, the maximum of resistance was decreased from cycle to cycle. The first heating–cooling cycle in the case of the ESD-TPU material was a setup cycle. In the case of the ESD-Onyx, the resistance had a maximum at around 20 °C. The ESD-PLA material was not suitable for this purpose.

The signal transfer capability of the composites was investigated. The results of the experiments showed that ESD-PLA, ESD-ABS, and ESD-TPU can be used as low-pass filters. Péntek and coworkers have found that similar conductive PLA and ABS composite materials could also be used as low-pass filters [19]. The ESD-Onyx composite is not suitable for electronic signal transmission due to its high attenuation, which was measured in all the layer height cases for this material.

Broken specimens of all materials tested were imaged by scanning electron microscopy. Comparing the results in the literature [43,44,45] and the SEM images of the ESD-ABS’ broken surface, the conductive filler of this material was carbon nanotube. This information was not included in the data published by the manufacturer.

Cell viability tests showed that none of the materials had cytotoxic effects on the cells. A slightly increased cell viability was observed in ESD-ABS, ESD-PLA, and ESD-TPU materials. The highest cell viability was observed in the case of ESD-PLA material, which is a well-known biocompatible and biodegradable plastic material that can potentially support cell viability. The biological properties indicate potential use cases for skin- or short-term contact devices such as sensors or electrodes in biomedical applications. However, before actual clinical use, a detailed characterization is essential in preclinical settings, according to the relevant international standards and ethical considerations.

In summary, composite polymer filaments with carbon-based additives have opened possibilities in low-voltage and medical applications. This study investigates mechanical, electrical, and cell viability properties of commercially available filaments with carbon-based fillers. Based on the results of the resistance measurements during cycle tensile tests and cyclical variation of temperature, as well as signal transmission capability, specimens manufactured from the ABS-based and TPU-based composites are suitable for sensors, such as temperature sensors, strain gauges, and low-voltage medical applications such as EEG and EMG sensors. Based on the results of cytotoxicity tests, low-voltage skin-contact sensors could be made from these materials, facilitating their medical use. The tested conductive materials based on ABS and TPU are appropriate for use as medical sensors, even in the simple form tested.

In terms of limitations, the tested materials were difficult to print. Some specimens were printed more than once due to printing errors. Another limitation of this study was that only one sample structure was examined. Other properties of this block structure (fill density etc.) or other structures should be investigated to improve the applicability of the composites for sensors.

## 5. Conclusions

Conductive polymers have extensive utilization in the topic of sensors and low-voltage applications. The study investigated the mechanical, electrical, and biological properties of four commercially available conductive polymer composites which incorporate carbon-based additives. The materials of the study were ESD-PLA, ESD-ABS, ESD-TPU, and ESD-Onyx. The tests performed were necessary to verify the suitability of the tested materials for low-voltage applications and use in medical sensors (e.g., temperature sensor, tactile sensor, and strain sensor).

Based on the results of the cyclic tensile tests, the ESD-ABS samples with 100 µm layer height could be used as a stress–strain stamp. These 100 µm layer height specimens have predictable behaviour in a cyclic tensile test. The resistance is unambiguous in the same stress and release branch. The change in resistance in one cycle does not exceed 50% in the 0.2 mm–0.8 mm strain interval, and this change can be called exponential. The gauge factor of these samples is between 40 and 120. Samples of this material with this simple structure have sufficient strain characteristics for use in a strain gauges. Strain sensor fabricated from the ESD-ABS conductive composite can be widely used in medical applications, e.g., prosthetics, ulcer detection, or strain detection in orthosis applications, but when fixing it should be noted that the first cycle is a “set-up cycle”.

According to the results of the resistance during flexural tests, the ESD-ABS composite can be used as a bending sensor if the bending angle is less than 18°.

Based on the cyclic temperature–resistance tests, the ESD-ABS and ESD-TPU composite materials are suitable for raw materials for thermometers and thermosensors, between 20 °C and 75 °C and 10 °C and 80 °C.

Based on the signal transfer capability measurements, the ESD-PLA, ESD-ABS, and ESD-TPU composites can be used as a low-pass filter. These materials can be used as electrocardiography (EEG) and electromyography (EMG) sensors, and in pulse oximeters with a combination of LED and SpO2 sensors to measure blood oxygen saturation. The ESD-Onyx composite is not suitable for use as a wire or other low-voltage electrical component. This material can only be used for ESD safe solutions, such as encapsulation.

According to the results listed, considering that none of the composites investigated are cytotoxic, the ABS-based and TPU-based composites could be suitable for use in a variety of medical sensors. Further studies should investigate strain, bending, and temperature sensors with structures other than those tested in this work using these materials. Strain sensors should be developed with special structures to improve the gauge factor. The further developed sensors could be used in medical sensors in clinical trials, mainly in the field of prosthetics and orthotics. Additionally, they could be considered for future application as an SMP (smart memory polymer) for 4D printing technology in biomedical use cases.

## Figures and Tables

**Table 1:** 3D printing parameters of the investigated materials.

Material	Nozzle Diameter [mm]	Hot-End Temperature [°C]	Printing Bed Temperature [°C]	Print Speed [mm/s]	Layer Height [μm]
ESD-PLA	0.4	210	70	30	100; 200; 300
ESD-ABS	0.4	245	110	10	100; 200; 300
ESD-TPU	0.8	250	55	30	100; 200; 300
ESD-Onyx	No data	No data	Unheated	No data	100; 125

**Table 2:** Tensile properties of the conductive materials.

Material	Young’s Modulus [MPa]	Tensile Strength [MPa]	Elongation at Tensile Strength [%]
ESD-PLA	1785.168 ± 34.451	22.856 ± 0.505	2.666 ± 0.207
ESD-ABS	1629.639 ± 24.656	20.432 ± 0.513	1.955 ± 0.028
ESD-TPU	63.721 ± 2087	4.981 ± 0.209	29.556 ± 1.733
ESD-Onyx	2006.854 ± 120.665	41.104 ± 1.017	13.606 ± 2.570

## Data Availability

All the measured data can be found in the following online data repository: Paári-Molnár, Emese; Kardos, Kinga; Told, Roland; Simon, Imre; Sahai, Nitin; Szabo, Peter; Bóvári-Biri, Judit; Steinerbrunner-Nagy, Alexandra; Pongracz, Judit; Rendeki, Szilard; Maroti, Peter (2024), “Dataset of Comprehensive study of Mechanical, Electrical and Biological Properties of Conductive Polymer Composites for Medical Applications through Additive Manufacturing”, Mendeley Data, V3, doi: 10.17632/6hgh5zrny5.3. In case of citing the data can be found in the “Supplementary Materials”, please, refer to the original article as well.

## Supplementary Materials

The following supporting information can be downloaded at: https://data.mendeley.com/datasets/6hgh5zrny5/3 (accessed on 14 September 2024).
